# Seasonal Levels of the *Vibrio* Predator *Bacteriovorax* in Atlantic, Pacific, and Gulf Coast Seawater

**DOI:** 10.1155/2013/375371

**Published:** 2013-12-22

**Authors:** Gary P. Richards, Michael A. Watson, E. Fidelma Boyd, William Burkhardt, Ronald Lau, Joseph Uknalis, Johnna P. Fay

**Affiliations:** ^1^United States Department of Agriculture, Agricultural Research Service, Dover, DE 19901, USA; ^2^Department of Biological Sciences, University of Delaware, Newark, DE 19716, USA; ^3^United States Food and Drug Administration, Gulf Coast Seafood Laboratory, Dauphin Island, AL 36528, USA; ^4^Kona Coast Shellfish LLC, Kailua-Kona, HI 96740, USA; ^5^United States Department of Agriculture, Agricultural Research Service, Wyndmoor, PA 19038, USA

## Abstract

*Bacteriovorax* were quantified in US Atlantic, Gulf, and Pacific seawater to determine baseline levels of these predatory bacteria and possible seasonal fluctuations in levels. Surface seawater was analyzed monthly for 1 year from Kailua-Kona, Hawaii; the Gulf Coast of Alabama; and four sites along the Delaware Bay. Screening for *Bacteriovorax* was performed on lawns of *V. parahaemolyticus* host cells. Direct testing of 7.5 mL portions of seawater from the Atlantic, Pacific, and Gulf coasts gave mean annual counts ≤12.2 PFU. Spikes in counts were observed at 3 out of 4 sites along the Delaware Bay 1 week after Hurricane Sandy. A comparison of summer versus winter counts showed significantly more *Bacteriovorax* (*P* ≤ 0.0001) in the Delaware Bay during the summer and significantly more (*P* ≤ 0.0001) in the Gulf during the winter, but no significant seasonal differences (*P* > 0.05) for Hawaiian seawater. *Bacteriovorax* counts only correlated with seawater salinity and temperature at one Delaware site (*r* = 0.79 and *r* = 0.65, resp.). There was a relatively strong negative correlation between temperature and *Bacteriovorax* levels (*r* = −0.585) for Gulf seawater. Selected isolates were sequenced and identified by phylogenetic analysis as *Bacteriovorax* clusters IX, X, XI, and XII.

## 1. Introduction

*Vibrio parahaemolyticus *and *Vibrio vulnificus *are important foodborne pathogens associated with the consumption of fish and shellfish, especially oysters, which have long been known to bioconcentrate vibrios within their edible tissues [1, 2]. *Vibrio vulnificus* also causes life-threatening illness from wound infections acquired in the marine environment [3]. Pathogenic vibrios show seasonal predilection in seawater and shellfish, with high counts during warmer months and low to negligible counts during the colder months [2, 4, 5]. Recently, we showed that naturally occurring *Bdellovibrio* and like organisms (BALOs) from coastal seawater significantly reduced the levels of *V. parahaemolyticus* and *V. vulnificus* in seawater and *V. parahaemolyticus* in seawater and oysters [6]. Among the BALOs are marine and terrestrial forms, with the marine forms associated with *Bacteriovorax, *which are exclusively saltwater predators [7, 8]. *Bacteriovorax* have shown preferential predation toward *V. parahaemolyticus* when compared to a broad range of potential host bacteria [9–12]. This suggests that *Bacteriovorax* may invade and kill *V. parahaemolyticus* in seawater more efficiently than other bacterial pathogens.

The life cycle of *Bacteriovorax* and other BALOs usually involve intracellular invasion of and replication within a host cell, although some are known to grow host-independently [13–16]. During the attack phase, BALOs propel themselves with a single polar flagellum to find a susceptible Gram-negative bacterium to serve as its host. The BALO enzymatically digests a hole in the host membrane, enters the periplasmic space, and, utilizing nutrients from the host, grows in a worm-like fashion in a structure known as a bdelloplast. When mature, the bdelloplast septates into multiple immature cells and are subsequently released from the host as it lyses. The immature cells develop into mature, attack phase cells to repeat the cycle all over again. Attack phase BALOs are small, with a diameter of only 0.2−0.4 *μ*m [16], making them filterable through 0.45 *μ*m pore size membranes, which facilitates their separation from host bacteria. Immature BALOs are about the same length but appear narrower and smoother than those in the attack phase [6], making them easily filterable as well. In natural unfiltered seawater spiked with >1 × 10^3^ CFU/mL *V. parahaemolyticus*, *V. parahaemolyticus* counts decreased by 3-logs to nearly nondetectable levels over 72 h, while naturally occurring BALOs (*Bacteriovorax*) increased by 3-logs from nearly nondetectable initial levels over this same period [6].

**Figure 1:**
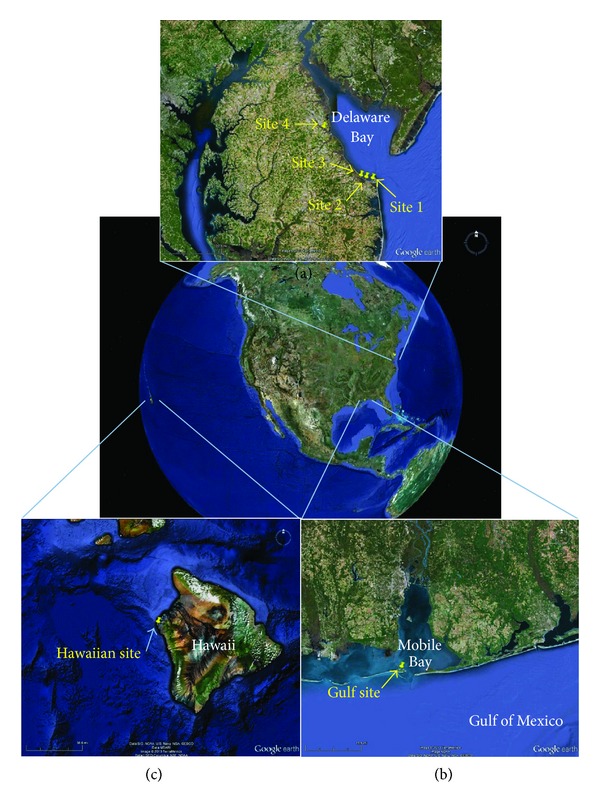
Sites of seawater collection along the (a) Delaware Bay, Delaware, (b) the Gulf Coast of Alabama, and (c) Keyhole Point near Kailua-Kona, Hawaii. Maps not to scale. Images were accessed through Google Earth.

Some studies have evaluated the seasonality of *Bacteriovorax* in marine systems including estuarine sediment [17] and seawater [18, 19]. The present study further evaluates the seasonality of *Bacteriovorax* among estuarine species. In this study, we evaluated natural seawater monthly for 1 year for total culturable *Bacteriovorax* that were capable of infecting *Vibrio parahaemolyticus* O3:K6 host cells (so called *Vibrio* predatory bacteria [6]) from four sites along the Delaware Bay (Figure 1(a)), one site from the Gulf Coast of Alabama (Figure 1(b)) and one site in Kailua-Kona, Hawaii (Figure 1(c)). Three of the collection sites along the Delaware Bay and the Gulf site were estuarine. We also identify seasonal patterns for *Bacteriovorax *abundance, evaluate the effects of temperature and salinity on *Bacteriovorax* levels, identify some of the *Bacteriovorax *phylotypes, and suggest environmental factors that may influence *Bacteriovorax* levels.

## 2. Materials and Methods

### 2.1. Bacterial Strains

A clinical strain of *V. parahaemolyticus* O3:K6 known as RIMD2210633 was used as host for the assay of *Bacteriovorax* from seawater. This pandemic strain was originally isolated from an airport quarantine station in Japan in 1996 and caused travelers' diarrhea [20, 21]. Stock cultures of this isolate were routinely grown in Luria Bertani (LB) broth (Beckton, Dickinson and Co., Sparks, MD) supplemented with 2% NaCl (3% NaCl total) or were streaked on LB agar (Beckton, Dickinson and Co.) with 2% added NaCl (3% NaCl total).

### 2.2. Sampling Sites

Atlantic seawater was surface water collected along the shoreline or rivers' edges during high tide and analyzed from four Delaware Bay sites as previously described [6]. In essence, the Delaware sites were Site 1: the Cape May-Lewes Ferry Terminal in Lewes, DE (38°46′57.85′′N; 75°07′04.73′′W); Site 2: the Broadkill River, outside the University of Delaware Marine Laboratory in Lewes, DE, 0.6 km upstream from the mouth of the river (38°47′26.37′′N; 75°09′51.36′′W); Site 3: Oyster Rocks Road boat landing on the Broadkill River in Milton, DE (38°48′08.01′′N; 75°12′11.57′′W); and Site 4: Scotton Landing on the Saint Jones River in Frederica, DE (39°05′05.94′′N; 75°27′39.99′′W) (Figure 1(a)). Seawater was immediately transported to the laboratory in an insulated cooler at ambient temperature. Seawater was also collected monthly from the dock of the U.S. Food and Drug Administration Laboratory at Dauphin Island, AL (30°15′25.57′′N; 88°6′21.73′′W) (Figure 1(b)), and shipped to Delaware overnight in an insulated cooler for testing. Seawater temperature and salinity were recorded for the Delaware and Gulf sites at time of water collection. Surface seawater was also provided by Kona Coast Shellfish Co., Kailua-Kona, HI (19°43′42.9′′N; 156°3′46.2′′W) (Figure 1(c)), and was express shipped in an insulated cooler to the laboratory where it was received within 2 days of collection. Temperature of the Hawaiian water at time of collection was 24-25°C and approximately 35 ppt salinity year round. The Hawaiian and Gulf water samples were shipped with ice packs during the summer; however, the ice and seawater were physically separated in order to prevent direct contact. After delivery to the laboratory, all seawater samples were analyzed within 4 h.

### 2.3. Plaque Assay

Counts of *Bacteriovorax* in 7.5 mL portions of unfiltered or 0.45 *μ*m filtered seawater were quantified on lawns of *V. parahaemolyticus* O3:K6 host cells grown in Pp20 agar (polypeptone peptone supplemented with Bacto agar), as recently described [6]. Preliminary studies showed that seawater filtration was necessary to reduce the levels of non-*Bacteriovorax* microbial contaminants that could otherwise overgrow the lawns of *V. parahaemolyticus* host cells, thus affecting the quantification of immature or attack-phase *Bacteriovorax*. Incubations were carried out at 22−26°C for 7 days. Potential bacteriophage plaques would be distinguished from *Bacteriovorax* plaques by their rapid, overnight formation; whereas, *Bacteriovorax* plaques require several days to form [17]. Although *Bacteriovorax *produce plaques on Pp20 agar, bacteriophages generally require more nutritionally complex media for plaque formation [17]. No bacteriophages were detected in this study. Since 7.5 mL of seawater was analyzed per assay, total culturable *Bacteriovorax* counts are listed as counts/7.5 mL seawater throughout this paper.

### 2.4. Scanning Electron Microscopy

Scanning electron microscopy was performed on select isolates as described previously [6] using a Quanta 200FEG microscope (FEI Co., Hillsboro, OR) after glutaraldehyde fixation, rinsing with 0.1 M imidazole buffer (pH 7.0), dehydration in graded ethanol rinses, critical point drying by vacuum (Denton, Cherry Hill, NJ), and sputter coating with gold in an argon atmosphere using an Edwards Scancoat Six sputter coater (West Sussex, United Kingdom).

### 2.5. Statistical and DNA Sequence Analyses

Correlation coefficients were obtained between *Bacteriovorax* counts and salinity or temperature, while significant differences in counts, temperatures, and salinities were determined by Student's *t* test. *P* values ≤ 0.05 were considered statistically significant while values ≤0.0001 were considered extremely statistically significant.

For 16S rRNA sequencing, PCR was performed on 13 randomly drawn isolates using 0.2 *μ*M final concentration of forward primer (Bac676F) and reverse primer (Bac1442R), as described by Davidov et al. [22], except there was no GC clamp on the 5′ end. Conditions were 94°C denaturation for 1 min followed by 45 cycles at 94°C for 1 min, 56°C for 1 min, and 72°C for 1 min. The cDNAs were electrophoretically purified on an 0.8% agarose gel and ethidium bromide-stained bands of ~700 bp were excised and purified using a QIAquick Gel Extraction kit (Qiagen #28704) following the manufacturer's instructions. Sequencing was performed by GENEWIZ, Inc. (South Plainfield, NJ) using primer Bac1442R. Data was trimmed at the terminal ends and sequences ~700 bp long were identified by GenBank Blast search and compared to phylotypes and clones described for similar sampling sites, as described by Pineiro et al. [9].

## 3. Results

**Figure 2:**
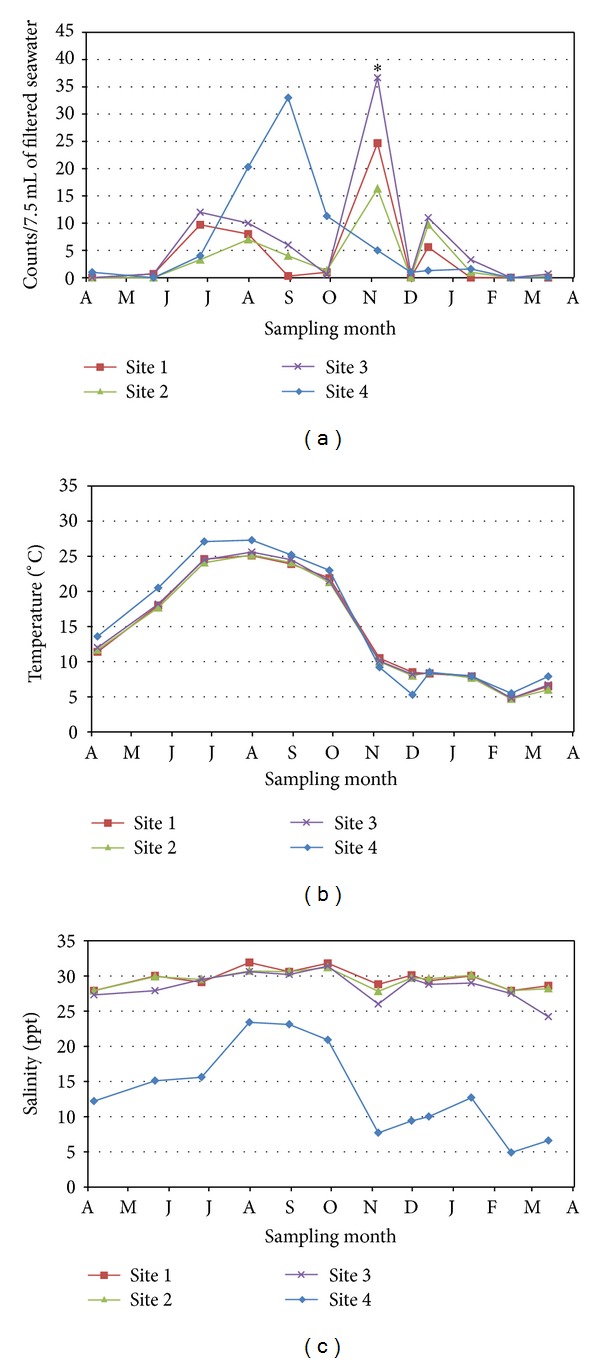
Monthly *Bacteriovorax* levels and water parameters at four Delaware sites over 1 year. (a) *Bacteriovorax* levels/7.5 mL seawater, (b) seawater temperature, and (c) seawater salinity. *Bacteriovorax* levels are the mean of three separate analyses for each site. The asterisk in (a) signifies *Bacteriovorax* counts from a sample collected 1 week after a major hurricane.

*Bacteriovorax* levels were determined for four sites along the Delaware Bay. One site (Site 1) was at the Cape May-Lewes Ferry Terminal near the mouth of the Delaware Bay, while the other three sites were on tidal rivers in close proximity to the Delaware Bay (Figure 1(a)). Mean monthly counts of *Bacteriovorax* per 7.5 mL of 0.45 *μ*m-filtered seawater were determined for 1 year and are shown in Figure 2(a). Water collection scheduled for late October 2012 was postponed because of Hurricane Sandy which impacted this area with strong tidal surge, hurricane-force winds, and extremely heavy rains during late October. The hurricane caused seawater collection for the October sampling to be delayed until November 5. *Bacteriovorax* counts were at their highest levels at three of the four collection sites one week after the hurricane (Figure 2(a), asterisk). The most inland site (Site 4) showed no increase after the hurricane but rather decreased from the previous month's mean count. Unlike the other sites, Site 4 experienced a dramatic increase in *Bacteriovorax* levels over the summer months, with a peak count of 33 PFU/7.5 mL seawater in early September. The *Bacteriovorax* also showed a seasonal predilection with significantly higher counts in the summer than in the winter (*P* ≤ 0.0001). *Bacteriovorax* were detected from at least one site in the Delaware Bay every month except in February 2013 when none (<0.33 PFU/7.5 mL seawater) were detected at any of the four collection sites. The lack of *Bacteriovorax* detection in February coincided with the lowest water temperatures (mean from 4 sites = 5°C) recorded during the year. Delaware Bay seawater temperatures over a 12-month period were similar among the four sites and showed normal seasonal fluctuations, ranging from lows of about 5°C in February and highs ≥25°C in late July (Figure 2(b)). Salinities were similar for Sites 1–3, but the mean salinity for Site 4 (Figure 2(c)) was significantly lower (*P* ≤ 0.0001) than the other sites.

**Figure 3:**
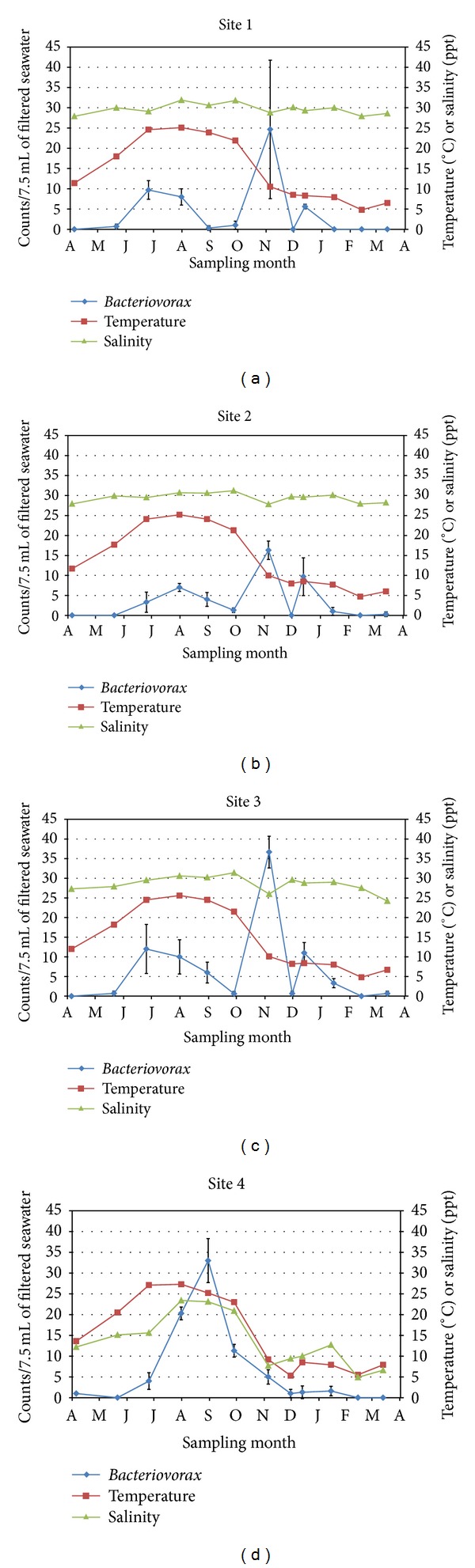
Overlay of *Bacteriovorax* level, temperature, and salinity for each of four sites along the Delaware Bay. ((a)–(d)) represent Sites 1–4, respectively. *Bacteriovorax* levels are the mean ± SD of three separate analyses for each site.

*Bacteriovorax* counts and corresponding temperatures and salinities are graphically overlaid for ease of comparison for each of the four sites in Figures 3(a)–3(d). Correlations between temperature and *Bacteriovorax* counts varied greatly between sites with *r* values of 0.102, 0.068, 0.053, and 0.651 for Sites 1−4, respectively. Site 4, the riverine site, was the most inland and showed a high correlation between temperature and *Bacteriovorax* counts. There was no correlation at the remaining sites. With one exception salinities remained >25 ppt throughout the year for three of the four sites (Figure 2(c)). Site 4 showed the lowest salinities throughout the study, ranging from a low of 4.8 ppt in February to a high of 23.4 ppt in late July (Figures 2(c) and 3(d)). In addition, there was little to no correlation between salinity and *Bacteriovorax* counts for Sites 1–3 with *r* values of −0.067, −0.119, and −0.183, respectively; however, there was a strong correlation between salinity and *Bacteriovorax* counts for Site 4 (*r* = 0.792). Spikes in counts were occasionally seen at each site (Figures 3(a)–3(d)) but were not consistently associated with temperature or salinity, except in the case of Sites 1–3, immediately after Hurricane Sandy where peaks in counts occurred as water temperatures dipped from >20°C the month before the storm to approximately 10°C after the storm (Figures 3(a)–3(c)).

Topographies of the Delaware sites differed somewhat. Site 1 was the southernmost site directly along a rock-fortified shoreline near the mouth of the Delaware Bay (Figure 1(a)). In contrast, the other three sites were tidal rivers with marsh grasses growing along the banks of the rivers. The presence or absence of marshes in the vicinity of the collection sites could not be shown to influence total *Bacteriovorax* levels over the course of this study. Site 4 showed a different trend in total *Bacteriovorax* counts from those of Sites 1–3, which were similar (Figure 2(a)). This may be due more to the fact that Site 4 was the greatest distance inland from the Delaware Bay (and had lower salinity) rather than to the presence or absence of marshes in the area.

**Figure 4:**
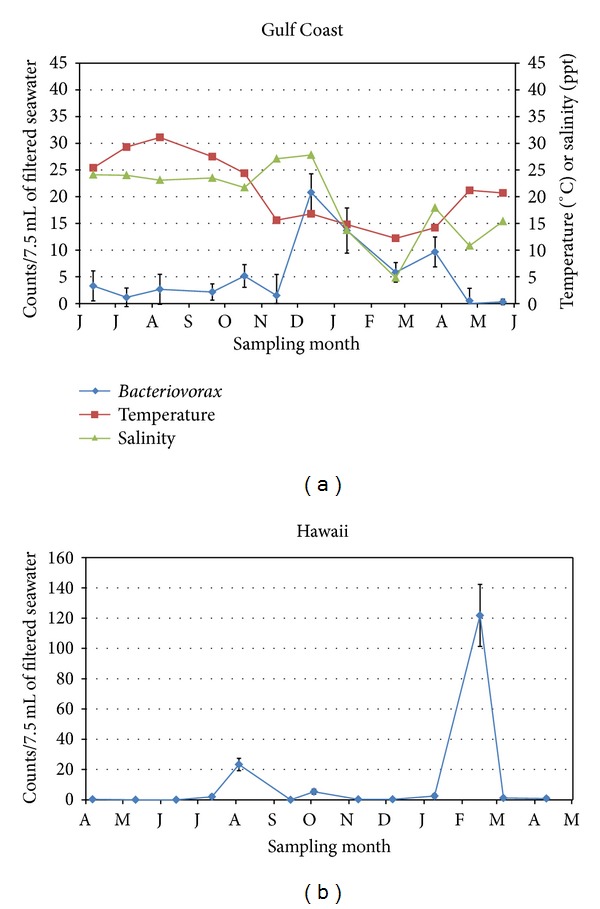
Monthly *Bacteriovorax* levels, seawater salinity, and temperature for (a) Gulf of Mexico site (starting in June 2012) for 1 year. (b) *Bacteriovorax* levels for Hawaiian site (starting in April 2012) for 13 months. Seawater temperature and salinity data are not shown for Hawaii because they remain steady between 24 and 25°C and at 35 ppt salinity. All monthly *Bacteriovorax* levels are the mean ± SD obtained from six samples.

Gulf Coast seawater ranged in salinities (4.8−28.8 ppt) and temperatures (12.2−31.1°C) (Figure 4(a)). In contrast to Delaware seawater, the Gulf Coast seawater showed low levels of *Bacteriovorax* (≤5 PFU/7.5 mL of seawater) throughout the summer months and significantly higher (*P* ≤ 0.0001) levels during the winter months (Figure 4(a)). This is opposite to the findings in Delaware, where significantly higher counts (*P* ≤ 0.0001) were obtained throughout the summer months. In fact, there was a relatively strong negative correlation between temperature and *Bacteriovorax* levels (*r* = −0.585) for Gulf seawater. The highest mean *Bacteriovorax* reading in the Gulf was obtained in December, concurrent with the highest salinity reading. As salinity levels dropped from January to February, so did the levels of total *Bacteriovorax* (Figure 4(a)). Nevertheless, correlation was low between salinity and *Bacteriovorax* counts over the course of the year (*r* = 0.211).

Hawaiian seawater, like the Delaware Bay and Gulf Coast seawater, was surface water, but it was not subjected to seasonal temperature changes or shifts in salinities. It had nearly constant temperature and salinity (24-25°C and 35 ppt) throughout the year. Results showed two spikes in *Bacteriovorax* counts, one in August followed by one in February, when mean counts increased from levels generally <5 PFU/7.5 mL of seawater to 23 and 121.8 PFU/7.5 mL of seawater, respectively (Figure 4(b)). The cause for these spikes remains uncertain. *Bacteriovorax* counts for both Gulf Coast and Hawaiian seawater may have been affected by shipping, which took 1 and 2 days, respectively. We cannot preclude the possibility that counts may have changed during transit, especially if potential host cells were present in the seawater. Predation on natural bacteria in the seawater could account for the two spikes in monthly counts that were observed for the Hawaiian seawater (Figure 4(b)).

Sequencing of isolates showed 99.9-100% identity (maximum of 1 base difference out of 700 bp per isolate) to comparable sequence submitted to GenBank by Pineiro et al. [9]. Phylotypes and clones are shown in Table 1 and indicate that the isolates were within four different phylotype clusters (IX, X, XI, and XII). Cluster XII isolates were clones of Coco2B and Hawaii5, as described previously [9]. None of the isolates were of the lower numbered clusters, like IV and V, which are more commonly believed to be low-salt, estuarine species [9, 19, 23]. Cluster IX isolates were from two sites in the Delaware Bay near Lewes, DE, and from one site along the Gulf Coast (Table 1). Previously, Pineiro et al. [9] obtained this same isolate from the Delaware Bay in Lewes and named it as clone Lewes11. The two Delaware isolates were obtained from a medium salinity site (Site 3) and a low salinity site (Site 4).

High levels of microbial contaminants were present in unfiltered Atlantic and Gulf Coast seawater, but Hawaiian seawater was cleaner, thus allowing a comparison of *Bacteriovorax *counts in unfiltered and 0.45 *μ*m filtered samples (Table 2). In unfiltered seawater, *Bacteriovorax* were higher for most of the months (Table 2). Only in August were the levels in unfiltered and 0.45 *μ*m filtered seawater essentially the same (23.5 versus 23.3 PFU/7.5 mL of seawater, resp.). In February 2013, counts in the unfiltered Hawaiian seawater were too numerous to count and far exceeded the 121.8 PFU/7.5 mL seawater that was observed for the filtered sample. These spikes in *Bacteriovorax* counts cannot be attributed to changes in temperature or salinity because temperature and salinity of the Hawaiian seawater remained nearly constant. Additionally, there were no unusual weather conditions to account for these high counts. Mean counts, excluding the February 2013 data, indicate the presence of 6.2 times more *Bacteriovorax* in unfiltered seawater than in filtered seawater.

**Figure 5:**
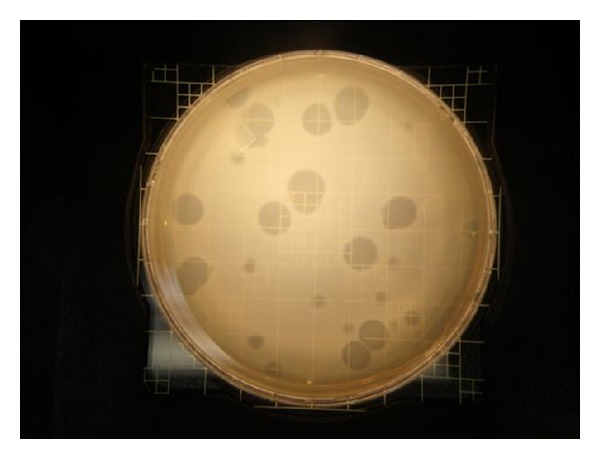
Plaques are readily visible from the assay of *Bacteriovorax* isolates on a lawn of *V. parahaemolyticus* host cells after incubation for 7 days. Larger plaques are approximately 1 cm in diameter in this image. Medium is Pp20 agar as indicated in Materials and Methods.

**Figure 6:**
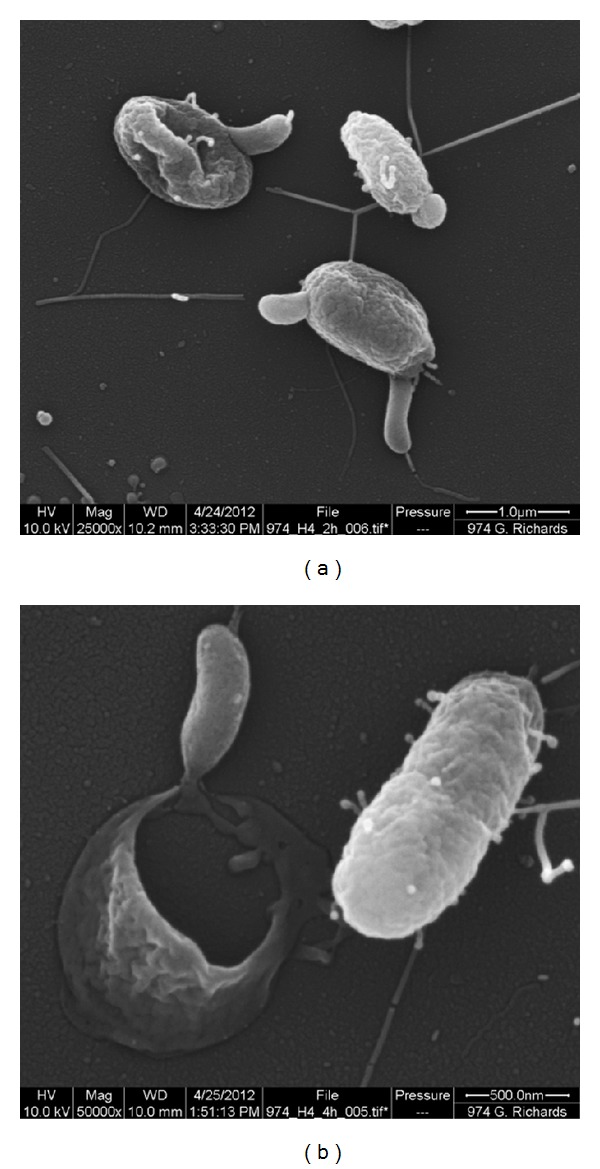
Scanning electron micrographs of *Bacteriovorax* infecting *Vibrio parahaemolyticus*. (a) Three vibrios shown with *Bacteriovorax *apparently entering (infecting) the *Vibrio* (lower right). The upper left cell shows a late-stage-infected *Vibrio* with the immature *Bacteriovorax* emerging from the cell. Note the appearance of the remaining wormlike *Bacteriovorax* within the partially shrunken *Vibrio*. (b) Immature stage *Bacteriovorax* (upper left) emerging from a dead *Vibrio*. Note the single hole in the *Vibrio* from which multiple immature *Bacteriovorax* would have emerged. An apparently uninfected *Vibrio* is shown on the right.

Representative plaques on Pp20 agar plates were picked to further evaluate some of the isolates. Plaque sizes often varied from one isolate to another and plaques were generally colorless (Figure 5). *Bacteriovorax* were usually isolated by picking from the center of large, well-isolated plaques; enriching in host cells suspended in sterile seawater; and imaging by scanning electron microscopy, as previously described [6]. A range of morphologies were observed, as shown in Figure 6, and included small, filterable forms (immature and attack-phase stages) and larger forms within the host (bdelloplast stage). Therefore, their ability to pass through 0.45 *μ*m filters depended on their stage in the life cycle. Areas of clearing around colonies were occasionally observed and were associated with the diffusion of inhibitory substances produced by the colonies. They were not counted as *Bacteriovorax* plaques. Sequencing of representative samples showed them to be *Pseudoalteromonas* spp. No rapidly forming plaques, representative of bacteriophages were observed during this study.

## 4. Discussion

Over the course of a year, *Bacteriovorax* were detected at the highest levels in the Delaware Bay during the summer and along the Gulf Coast during the winter. This latter finding may be surprising at first, since *Vibrio* levels are typically very low during the winter months. However, *Bacteriovorax* are known to have broad host specificities against a wide variety of Gram-negative bacteria [9–12]. Although this study concentrated on culturable *Bacteriovorax* that infected *V. parahaemolyticus* and hence may be referred to as *Vibrio* predatory bacteria [6], it would be expected that the *Bacteriovorax* likely preyed on many other Gram-negative bacteria outside the *Vibrionaceae* family, particularly when *Vibrio* levels were low. *Bacteriovorax* were isolated nearly year round from small volumes (7.5 mL) of seawater without the need for enrichment. It should be recognized that the above counts represent the number of culturable *Bacteriovorax* in 0.45 *μ*m-filtered seawater and may underrepresent the total number of *Bacteriovorax* within the water. Figure 6 shows the relative size of *Bacteriovorax* compared to its *Vibrio parahaemolyticus* host. Although the immature and attack-phase *Bacteriovorax* are small enough to pass through a 0.45 *μ*m filter, an appreciable number of *Bacteriovorax* might not; thus counts expressed throughout this paper should be considered as minimal counts derived principally from immature and attack phase *Bacteriovorax*. Larger *Bacteriovorax*, that is, those maturing as bdelloplasts within the host cell or the so-called “host independent” strains that occasionally grow as elongated chains, would readily be filtered out. By comparing levels of *Bacteriovorax* in filtered and unfiltered Hawaiian seawater (Table 2), we estimate that 84% of the *Bacteriovorax* in seawater were unable to pass through a 0.45 *μ*m filter. This highlights a shortcoming of using filtered seawater for the quantification of *Bacteriovorax* in culture-dependent methods. It is uncertain if season has any influence on the prevalence of different life forms or sizes of *Bacteriovorax*.

The presence of a variety of *Bacteriovorax* phylotypes having different preferences for both salinity and temperature was reported in a study of the Cheaspeake Bay where some phylotypes (clusters IV and V) were low to medium salinity (estuarine) species, whereas others (clusters IX and XII) were recovered from medium or high salinity sites [9, 23]. Sequence analysis of 13 samples in this present study showed that all the isolates were in clusters IX, X, XI, or XII (Table 1), regardless of the isolates' origin. As mentioned previously, four of the sequenced isolates obtained from the lowest salinity site in Delaware (Site 4) were clusters IX, X, or XII—clusters more commonly associated with higher salinity seawater [23]. In the present study, salinities did not deviate greatly for three of the Delaware Bay sites or for seawater obtained from Hawaii; however, seawater from the Gulf Coast and from Delaware Bay Site 4 varied appreciably. When the salinities were the highest at these two sites, the *Bacteriovorax* levels peaked (Figures 3(d) and 4(a)). We showed that salinity and total *Bacteriovorax* counts correlated well (*r* = 0.792) at the lowest salinity Delaware Bay site (Site 4) but not at the higher salinity Delaware sites. Compared to the other sites, Site 4 had relatively low and widely varying salinities; therefore, the strong correlation between salinity and *Bacteriovorax *counts at this site must be tempered by the fact that this riverine location never reached the higher salinity levels found in the other Delaware Bay sites. Pineiro et al. detected clusters IV and V in low to moderate salinity areas of the Delaware Bay and in the nearby Chesapeake Bay [23]. We did not identify any cluster IV or V isolates in Delaware or elsewhere, even though these clusters are considered more estuarine species [9, 23]. Part of the reason may be that our sampling was very limited and was not intended to be a comprehensive analysis of phylotypes, since the dynamic mixing of various proportions of fresh or low-salinity water and seawater in these areas during high tide would be expected to continually alter the phylotype profiles. The identification of only phylotypes IX, X, XI, and XII in the Delaware Bay does not preclude the presence of other undetected phylotypes from among the many unsequenced *Bacteriovorax* that were enumerated over the year.

Studies have demonstrated that relaying of oysters to higher salinity areas reduces *Vibrio* levels faster than when oysters are maintained in lower salinity waters [24, 25]. High salinity relay has been proposed as a postharvest processing strategy to reduce *V. vulnificus* in oysters and was also shown effective in eliminating *V. parahaemolyticus* as well [26]. The mechanism by which high salinity regimes enhance *Vibrio* reductions in oysters remains uncertain. We recently hypothesized that high salinity supports the proliferation of predatory bacteria and that drought conditions in North Carolina from 2007 to 2009 may have been responsible for the apparent disappearance of *V. vulnificus* from seawater and oysters [6], as reported by Froelich et al. [27]. Other factors beyond salinity and temperature that may well affect *Bacteriovorax* levels and phylotypes within rapidly changing estuarine systems include dramatic fluctuations in flow rates due to storm events, currents and winds, runoff, physical and chemical parameters of the seawater, and perhaps proximity to marshlands, beaches, farmlands, forested or residential areas, or rocky shorelines.

The amount of nutrients and suspended solids in the water varied and resuspended sediment was commonly observed. During the winter, *Bacteriovorax* could not be found in top or bottom water samples from the Chesapeake Bay but were isolated during this period from sediment [23]. This may indicate that greater numbers of *Bacteriovorax* are present in the sediment in the winter and perhaps at other times of the year; therefore, resuspension of sediment into the water column might influence the *Bacteriovorax* levels detected in the water. Our finding that seawater taken from the Delaware Bay one week after Hurricane Sandy contained the highest levels of *Bacteriovorax* detected in the Delaware waters may be due to the resuspension of large amounts of sediment into the water column by the hurricane. These are all factors that affect shellfish growing areas and the levels of *Bacteriovorax* within these areas. The combination of these highly variable factors will complicate full discernment of the intricacies of predatory interactions with vibrios and other bacteria in the environment.

Oysters and other molluscan bivalve shellfish are well known for filtering pathogens and other contaminants from seawater making shellfish potential sources for illness. *Bacteriovorax* that may be suspended in seawater or attached to suspended matter are likely sources of food for bivalve shellfish and could concentrate within the shellfish similar to that of pathogens. Tests for *Bacteriovorax* quantification within shellfish tissues have not been developed to date; however, we hypothesize that *Bacteriovorax* within shellfish would continue to parasitize vibrios and other potential pathogens much as they do in seawater, thus rendering shellfish safer to consume when they are obtained from areas where *Bacteriovorax* are present in higher numbers. Our previous work indicates that *V. parahaemolyticus* levels drop significantly in oysters and seawater as *Vibrio* predatory bacteria increase in the surrounding water [6]. Our objectives over the next few years are to (a) develop methods to accurately quantify *Bacteriovorax* levels within oyster tissues, (b) determine the relationships between *Bacteriovorax* and *Vibrio* counts in seawater and in shellfish, and (c) develop potential commercial processing strategies using *Bacteriovorax* to reduce vibrios and other potential pathogens in shellfish.

## Figures and Tables

**Table 1:** Phylotypes of selected *Bacteriovorax* isolated from seawater.

Cluster	Strain/isolate^1^	Origin^2^	Isolate designation
IX	Lewes11	Gulf Coast, Alabama	G3
		Delaware Site 3	OR7
		Delaware Site 4	S12
X	Hawaii	Delaware Site 3	OR3
		Delaware Site 4	S13
		Delaware Site 4	S15
XI	Tri101	Delaware Site 3	OR1
XII	Coco2B	Delaware Site 3	OR2
		Delaware Site 2	OS1
		Delaware Site 2	OS2
		Delaware Site 4	S11
XII	Hawaii5	Hawaii	H4
		Hawaii	H8

^1^Strain designation used in Pineiro et al. [9].

^2^See site map in Figure 1.

**Table 2:** Comparison of *Bacteriovorax* levels in unfiltered versus 0.45 *μ*m filtered Hawaiian seawater. Each monthly count is the mean of six replicate assays.

Date	Unfiltered seawater (PFU/7.5 mL)	Filtered seawater (PFU/7.5 mL)
2012		
April 6	9.4	0.3
May 11	6.3	0.0
June 13	17.5	0.0
July 12	14.2	2.0
Aug. 3	23.5	23.3
Sept. 14	16.5	0.0
Oct. 3	17.8	5.3
Nov. 8	47.8	0.3
Dec. 6	23.5	0.3
2013		
Jan. 9	20.7	2.5
Feb. 13	TNTC (>125)^1^	121.8^1^
Mar. 6	19.5	1.2
Apr. 13	7.5	0.8
Mean^1^:	18.7	3.0

^1^Abbreviation: TNTC: too numerous to count. Mean counts exclude the February 13, 2013 data.
